# Retroperitoneal Lymph Node Dissection as Primary Treatment for Metastatic Seminoma

**DOI:** 10.1155/2018/7978958

**Published:** 2018-02-01

**Authors:** Brian Hu, Siamak Daneshmand

**Affiliations:** ^1^Department of Urology, Loma Linda University, 11234 Anderson Street Room A-560, Loma Linda, CA 92374, USA; ^2^University of Southern California Institute of Urology, 1441 Eastlake Avenue, Los Angeles, CA 90033, USA

## Abstract

Reducing the long-term morbidity in testicular cancer survivors represents a major area of interest. External beam radiation therapy and systemic chemotherapy are established treatments for seminoma; however, they are associated with late toxicities such as cardiovascular disease, insulin resistance, and secondary malignancy. Retroperitoneal lymph node dissection (RPLND) is a standard treatment for nonseminomatous germ cell tumors (NSGCT) that has minimal long-term morbidity. Given the efficacy of RPLND in management of NSGCT, interest has developed in this surgery as a front-line treatment for seminoma with isolated lymph node metastasis to the retroperitoneum. Four retrospective studies have shown promising results when surgery is performed for seminomas with low-volume retroperitoneal metastases. To better determine if RPLND can be recommended as a primary treatment option, two prospective clinical trials (SEMS and PRIMETEST) are underway. This review will examine the literature, discuss the benefits/limitations of RPLND, and compare the methodologies of the two ongoing clinical trials.

## 1. Introduction

Seminoma with isolated retroperitoneal lymphadenopathy is typically treated with external beam radiation therapy (XRT) or systemic chemotherapy. There has been little change in these recommendations over the last few decades. However, evidence continues to mount with regard to the long-term morbidities associated with these treatments. The risk of secondary malignancies is approximately twofold higher in patients who have had either chemotherapy or XRT for management of germ cell cancers [1]. The risk of cardiovascular disease is also high with testicular cancer survivors having up to a 2.6-fold increased risk over 20 years. Importantly, these long-term toxicities have been linked to decreases in overall survival [2, 3]. Other side effects can include lung injury, metabolic syndrome, renal toxicity, and decreases in fertility. As most testicular cancer survivors will live many decades, the impact and incidence of these toxicities can be profound.

There has been a concerted emphasis to reduce treatment-related morbidity in testicular cancer. A greater utilization of active surveillance in stage I disease, decrease in radiation dosage, limitations in the fields of radiation, and single-agent chemotherapy are examples of efforts to mitigate long-term toxicities. In line with this philosophy, investigators have looked to surgery for treatment of low stage metastatic seminoma given its effectiveness in treating germ cell tumors.

## 2. Rationale for Retroperitoneal Lymph Node Dissection

Retroperitoneal lymph node dissection (RPLND) represents an attractive treatment option for metastatic seminoma mainly because of the surgery's well-established efficacy. In seminoma, RPLND is generally recommended for residual retroperitoneal masses >3 cm following risk-adapted chemotherapy. In nonseminomatous germ cell tumors (NSGCT), RPLND is a treatment option for patients with high-risk stage I disease and for residual retroperitoneal masses ≥1 cm following systemic chemotherapy for metastatic disease [4]. Importantly, it can be also the primary treatment for stage IIA NSGCT with negative serum tumor markers. Not only is the surgery therapeutic, but it offers accurate pathologic staging with up to 30% of patients with stage I NSGCT having occult metastases and up to 35% of patients with clinical stage IIA disease being downstaged to stage I disease [5].

There are several other reasons that make RPLND a logical treatment for seminoma. A major reason why the surgery has proven to be effective is because of the predictable pattern of lymphatic spread of germ cell cancers. Given that pure seminoma lacks choriocarcinoma, the histology known to spread hematogenously, this could theoretically make RPLND for seminoma even more efficacious. Additionally, physicians treating testicular cancer are already familiar with the procedure and the surgical morbidity continues to decrease. Template dissections and nerve-sparing approaches are established methods for preventing retrograde ejaculation. Newer techniques with laparoscopy or a midline, extraperitoneal approach can also minimize morbidity including decreases in blood loss and length of hospitalization [6, 7].

Lastly, XRT and chemotherapy have limitations. For example, patients with a horseshoe kidney, inflammatory bowel disease, or a history of radiotherapy are not good candidates for XRT. Those with renal insufficiency or pulmonary disease could be precluded from effective chemotherapy. In these cases, another treatment option could prove invaluable.

## 3. Retrospective Data

There have been four published studies that evaluate RPLND as a primary treatment for testicular seminoma (Table 1). The first study was reported by Warszawski and Schmucking in 1997 from Germany [8]. This study retrospectively reviewed the results of 63 patients with stage I and II seminoma after RPLND (*n* = 63) from 1975 to 1985 and compared the results with patients who received XRT. Most patients had stage I seminoma (*n* = 45), though some had stage IIA (*n* = 7), IIB (*n* = 6), and IIC (*n* = 5) disease.  Table 2 provides a review of stage II seminoma TNM staging [9].

In those with clinical stage I seminoma, there was a 17.5% incidence of occult retroperitoneal disease, in line with current relapse rates seen in surveillance series. In patients with stage II seminoma, 6.3% were downstaged. In patients with stage I or IIA seminoma, with a median follow-up of 79 months, there was a 5.7% recurrence rate. The surgery provided excellent regional control with all the recurrences being identified as out of the retroperitoneal field. The efficacy of RPLND with larger nodal disease (>2 cm) decreased, with 6/11 (55%) patients recurring in the retroperitoneum.

Though there was no statistical difference in recurrence rates or actuarial survival when comparing XRT to RPLND, the authors concluded that results of XRT “seem to be superior.” One reason the authors cited was that the in-field recurrence rate was lower after XRT. When closely examining this, the recurrence rates varied drastically when stratified by clinical stage. Importantly, there were no in-field recurrences after RPLND for stage I and IIA seminoma, which was the same for XRT.

The first of the three more modern studies was by Mezvrishvili and Managadze [10]. They evaluated the outcomes of ten patients with high-risk stage I seminoma and four patients with stage IIA disease. Of the patients with stage I seminoma, there were three (30%) with retroperitoneal metastases at the time of surgery. All patients with clinical lymph node metastases had confirmation of disease after RPLND, and none underwent adjuvant treatment. With a mean follow-up of 56 months, they did not have any cases with local or distant recurrence.

Our group has reported on the outcomes of four patients with pure testicular seminoma after RPLND [11]. Three patients had clinical stage IIA seminoma, and one patient had clinical stage IIC disease, with a lymph node 5.5 cm in size. This patient had a presumed burned out primary tumor with scar with dystrophic calcification on the orchiectomy specimen. Patients underwent an open, modified-template RPLND through a midline, extraperitoneal approach [12]. All patients were discharged home on postoperative day 3. Three patients had pathologic stage IIA disease, and one had stage IIB due to a 2 cm lymph node with extranodal extension. No patients underwent adjuvant therapy. With a median follow-up of 25 months, there were no recurrences or deaths.

Lastly, Lusch et al. from Germany have recently presented a series on open or robotic RPLND in patients with stage IIA/B seminoma [13]. They identified 11 patients who underwent RPLND. Three of these patients (22%) received one cycle of carboplatin prior to RPLND. With a mean follow-up of 18 months, they had a 36% recurrence rate. One of the patients with recurrence had more advanced disease with clinical stage IIC disease, an initial lymph node metastasis >6 cm, and a clinically positive inguinal lymph node. All patients who recurred were salvaged with radiotherapy and chemotherapy, and 3 out of 4 have no evidence of disease.

Taken together, these studies include a total of 92 patients with stage I-IIC seminoma and 14 who experienced recurrence. The overall recurrence rate for all patients was 14% with patients having higher stage disease being at greater risk of recurrence.

## 4. Clinical Trials

This retrospective data has established promising oncologic benefit of RPLND in early stage seminoma. There are currently two active prospective clinical trials formally evaluating the efficacy of the surgery (Table 3).

### 4.1. SEMS

Our group has started the SEMS (Surgery in Early Metastatic Seminoma) trial, which is a multiinstitutional phase II trial of primary RPLND to treat testicular seminoma with isolated retroperitoneal metastases [14]. The main inclusion criteria are testicular seminomas with the presence of at least one retroperitoneal lymph node between 1 and 3 cm in size. No more than two lymph nodes can be clinically positive. Serum tumor markers may be mildly elevated. The lymphadenopathy can be identified at diagnosis or can represent recurrence in a patient originally diagnosed with stage I seminoma. The recurrence must be within 3 years of the cancer diagnosis in order to avoid enrolling those with late relapse that may represent a different biology.

The trial is currently open and accruing at 9 sites in the United States (University of Southern California, Loma Linda University, University of California San Francisco, Emory University, University of Chicago, Indiana University, Johns Hopkins, Mayo Clinic, and University of Oklahoma). The study has a primary endpoint of recurrence-free survival at 2 years. Secondary endpoints are recurrence-free survival at 5 years, percent of patients who can avoid XRT or systemic chemotherapy, and the complication rate of RPLND (short and long term). The estimated enrollment is 46 with a planned study completion date in the year 2020.

A clinical correlation in this study is utilization of PET scanning preoperatively. Though the established role of PET scanning in germ cell cancer is in postchemotherapy seminoma, it is often utilized in earlier stage disease. Patients undergoing RPLND will have a PET/CT scan done prior to surgery. These results will be compared with intraoperative lymph node pathology and may determine if this imaging modality has any utility in seminoma prior to chemotherapy.

### 4.2. PRIMETEST

The second study is PRIMETEST (Trial to Evaluate the Progression Free Survival with Primary Retroperitoneal Lymph-Node Dissection pRPLND Only in Patients with Seminomatous Testicular Germ Cell Tumors with Clinical Stage IIA/B) [15]. This study is based out of Heinrich-Heine University in Duesseldorf, Germany, and includes patients with testicular seminoma and retroperitoneal or inguinal lymphadenopathy with a maximum size of 5 cm. Only patients with unilateral disease are included in the study. The study includes those with multiple metastases as long as none is >5 cm. This trial also includes patients who experienced recurrence after a single dose of carboplatin chemotherapy.

Patients will undergo a modified-template RPLND, which can be done in the open fashion or laparoscopically with robotic assistance. The primary endpoint is progression-free survival at 3 years, and the study was designed to exclude a recurrence rate of >30% compared with standard treatment. Secondary endpoints include overall survival, complication rates, quality of life, long-term sequelae, and the rate of retrograde ejaculation. The study plans to accrue 30 patients with an estimated study completion of June 2021.

## 5. Limitations and Safety of RPLND

Given that RPLND for germ cell tumors have been performed since the early 1900's, the short- and long-term risks have been well documented [16, 17]. The long-term effects of the surgery include retrograde ejaculation, incisional hernia, and bowel obstruction. Most of the risk of surgery is associated with short-term complications including injury to retroperitoneal or peritoneal structures, ileus, bowel obstruction, chylous ascites, thromboembolism, and infection. We recently reported outcomes of our midline extraperitoneal approach to RPLND with no cases of ileus noted in 68 consecutive cases [6].

Some have expressed concern regarding the surgical planes with seminoma. The desmoplastic reaction after chemotherapy in seminoma can be intense and greatly increase the morbidity and technical difficulty of the surgery. This is secondary to the significant fibrosis that is seen with treatment of metastatic seminoma. However, from personal experience and reports from other surgeons who have performed these surgeries, the surgical planes in a primary RPLND for untreated seminoma are the same as would be encountered in NSGCT.

## 6. Managing Pathology after RPLND

A major benefit from surgery is that pathology can help inform management decisions. Ideally, RPLND will cure a large majority of patients while identifying those at high risk of recurrence. The high-risk patients can then be directed towards adjuvant treatments to further reduce recurrences. Factors such as lymph node positive count, lymph node size, and extranodal extension could become important in risk stratification.

In general, patients will fall into one of three categories after RPLND: those with more favorable pathology, those with the same pathology, and patients who have worse disease than anticipated. Those who are downstaged (e.g., stage I seminoma) could be placed on a less rigorous surveillance schedule. For the other two scenarios, it is important that the reasoning behind surgery be delineated early. In the SEMS trial, the rationale for RPLND is to give patients the opportunity to completely avoid XRT and chemotherapy, which is one of the secondary endpoints. This is the major reason why a ≤3 cm lymph node size was chosen. In patients with nonbulky lymphadenopathy, the data demonstrates that RPLND has a good chance of cure without adjuvant treatment. Therefore, if the pathology matches with the clinical stage, we feel that surveillance should be encouraged. However, in cases of upstaging, adjuvant treatment with chemotherapy can be considered. Chemotherapy is favored over XRT because chemotherapy can treat systemic disease and is preferred for higher stage disease.

The rationale behind the RPLND in the PRIMETEST trial is slightly different. This study hypothesizes that the 3-4 courses of chemotherapy for stage IIA or IIB seminoma is overtreatment. The investigators have selected a larger lymph node size of up to 5 cm, which will likely result in a higher recurrence rate. However, the investigators also hypothesize that a single, adjuvant dose of chemotherapy will reduce the recurrence risk with minimal long-term morbidity. If the recurrence rate from surgery is less than 30%, the investigators feel justified that RPLND with a short course of adjuvant chemotherapy will reduce morbidity. Additionally, they plan future studies to determine which patients can undergo surveillance and who should preferentially receive chemotherapy.

## 7. Conclusions

There are many reasons why RPLND represents a logical treatment for seminoma metastatic to the retroperitoneum. To date, there have been four retrospective studies that have shown promising results when RPLND is utilized as a primary treatment for early metastatic seminoma. As would be expected, recurrence rates seem to increase with larger retroperitoneal metastases. There are two active phase II clinical trials evaluating the recurrence-free survival of patients after a primary RPLND. The SEMS trial is multiinstitutional effort in the United States that includes patients with lymph nodes 1–3 cm in size. The PRIMETEST trial from Germany includes patients with lymph nodes <5 cm in size. The results of these studies will help determine if patients with metastatic seminoma will have a treatment option with minimal long-term morbidity.

## Figures and Tables

**Table 1:** Series of RPLND as primary treatment for seminoma.

Study	*n*	Stage	Type of RPLND	Discordant staging	Recurrence rate	Follow-up
Warszawski et al. [8]	63	I (*n* = 45) IIA (*n* = 7) IIB (*n* = 6) IIC (*n* = 5)	Open	24% 17.5% upstaged 6.3% downstaged	14% Stage I: 7% Stage IIA: 0% Stage IIB: 67% Stage IIC: 40%	79 mo
Mezvrishvili et al. [10]	14	I (*n* = 10) IIA (*n* = 4)	Open, nerve sparing	21% (all upstaged)	0%	56 mo
Hu et al. [11]	4	IIA (*n* = 3) IIC (*n* = 1)	Open, midline extraperitoneal, nerve sparing	50% 25% upstaged 25% downstaged	0%	25 mo
Lusch et al. [13]^∗^	11	IIA and IIB	Open and robotic, nerve sparing	Not described	36%	18 mo

^∗^Abstract.

**Table 2:** Stage II seminoma.

IIA	Any pT/Tx	N1	M0	S0 or S1
IIB	Any pT/Tx	N2	M0	S0 or S1
IIC	Any pT/Tx	N3	M0	S0 or S1

cN1 = metastases to single or multiple retroperitoneal lymph nodes ≤2 cm in size; cN2 = metastases to single or multiple retroperitoneal lymph nodes 2–5 cm in size; cN3 = metastases to single or multiple retroperitoneal lymph nodes >5 cm in size; pN1 = metastases to single or multiple retroperitoneal lymph nodes ≤2 cm in size, no more than 5 positive lymph nodes; pN2 = metastases to single or multiple retroperitoneal lymph nodes 2–5 cm in size, metastases to >5 lymph nodes with none >5 cm in size, extranodal extension; pN3 = metastases to single or multiple retroperitoneal lymph nodes >5 cm in size. Used with the permission of the American Joint Committee on Cancer (AJCC), Chicago, Illinois. The original source for this material is the AJCC Cancer Staging Manual, seventh edition (2010) published by Springer Science and Business Media LLC, www.springer.com.

**Table 3:** Prospective clinical trials of RPLND in seminoma.

	SEMS (Surgery in Early Metastatic Seminoma)	PRIMETEST (Trial to Evaluate Progression Free Survival with Primary Retroperitoneal Lymph-Node Dissection (pRPLND) Only in Patients with Seminomatous Testicular Germ Cell Tumors with Clinical Stage IIA/B)
Phase	II	II
Inclusion criteria	Testicular seminoma Retroperitoneal *lymph node 1–3 cm in size* No more than two enlarged lymph nodes	Testicular seminoma Inguinal, paraaortic, or retroperitoneal lymph nodes classified as local or regional unilateral metastasis Maximum dimensions of *lymph node metastasis 5 cm* Allow patients who have received single dose carboplatin for stage I seminoma
Exclusion criteria	Second primary malignancy History of radiation/chemotherapy	Prior scrotal or retroperitoneal surgery History of radiation/chemotherapy (other than carboplatin)
Serum tumor markers	Beta-HCG normal Allow LDH and AFP up to 1.5 times upper limit of normal	Exclude AFP elevation suspicious for NSGCT
Primary endpoint	2-year recurrence-free survival	3-year progression-free survival
Secondary endpoints	5-year recurrence-free survival Treatment-free survival (time free from radiotherapy or chemotherapy) Complication rate (long and short term)	Overall survival Quality of life Complication rate Long-term sequelae
Accrual goal	46	30
Start date	August 2015	June 2016
Target completion date	August 2020	June 2021
Number of institutions	9	1
Primary location	University of Southern California	Department of Urology, Heinrich-Heine University, Duesseldorf
Principal investigator	Siamak Daneshmand	Peter Albers
